# P16 COVID-19 and urinary tract infection: the main manifestations and their management

**DOI:** 10.1093/jacamr/dlad077.020

**Published:** 2023-08-02

**Authors:** Kiarina Chichirelo-Konstantynovych, Tetyana Konstantynovych

**Affiliations:** National Pirogov Memorial Medical University, Vinnytsya, Ukraine; National Pirogov Memorial Medical University, Vinnytsya, Ukraine

## Abstract

**Background:**

Despite the predominant injury from COVID-19 being to the respiratory system, there are presently several reports of urinary symptoms. The majority of them are due to comorbid urinary tract infection (UTI) but separate clinical signs can be related to the COVID-19 origin.

**Objectives:**

To investigate the frequency of UTI symptoms among patients recovering from COVID-19.

**Subjects and Methods:**

A total of 150 patients (males, 49.3%; average age 57 years old) at the average period of recovering after COVID-19 (6–7 months) were asked about quality, frequency and severity of the main UTI symptoms and antibiotics prescription for their treatment.

**Results:**

Sixty-one (40.7%) of the individuals asked reported anamnestic UTI (nephritis, glomerulonephritis, urolithiasis) prior to COVID-19 infection. One hundred and nine (72.6%) patients mentioned the appearance of UTI symptoms after recovering from COVID-19 (50% of them observed complaints in the period 3–4 months after hospital discharge). The prevalence of urinary symptoms looked as follows: haematuria, 17.43%; nocturia, 27.25%; incomplete emptying, 19.27%; frequent urination, 11.93%; leukocyturia, 24.12%. Forty-four patients (40.36%) visited family doctors for consultation regarding urinary complaints. Thirty-seven patients (33.94%) were recommended antibiotic therapy according to UTI diagnosis.

**Conclusions:**

Lower urinary tract symptoms are highly prevalent among COVID-19 patients. A possible reason is ACE-2, an essential receptor for the SARS-CoV-2 spike protein. COVID-19 can worsen previous chronic urinary tract pathologies and increase the risk of new infection, thereby creating the need for additional antibiotics.

